# PluriBAC: A Versatile Baculovirus-Based Modular System to Express Heterologous Genes in Different Biotechnological Platforms

**DOI:** 10.3390/v15101984

**Published:** 2023-09-23

**Authors:** Leslie C. Amorós Morales, Abril Marchesini, Santiago M. Gómez Bergna, Matías García Fallit, Silvana E. Tongiani, Larisa Vásquez, María Leticia Ferrelli, Guillermo A. Videla-Richardson, Marianela Candolfi, Víctor Romanowski, Matías L. Pidre

**Affiliations:** 1 Instituto de Biotecnología y Biología Molecular (IBBM, UNLP-CONICET), Facultad de Ciencias Exactas, Universidad Nacional de La Plata, Consejo Nacional de Investigaciones Científicas y Técnicas, La Plata B1900, Argentina; amorosleslie@biol.unlp.edu.ar (L.C.A.M.); abrilmarchesini@biol.unlp.edu.ar (A.M.); sgomezbergna@biol.unlp.edu.ar (S.M.G.B.); stongiani@biol.unlp.edu.ar (S.E.T.); larisavasquez@biol.unlp.edu.ar (L.V.); lferrelli@biol.unlp.edu.ar (M.L.F.); victor@biol.unlp.edu.ar (V.R.); 2 Instituto de Investigaciones Biomédicas (INBIOMED, UBA-CONICET), Facultad de Medicina, Universidad de Buenos Aires, Consejo Nacional de Investigaciones Científicas y Técnicas, Ciudad Autónoma de Buenos Aires C1121A6B, Argentina; mati.garciafallit@gmail.com (M.G.F.); mcandolfi@fmed.uba.ar (M.C.); 3 Fundación Para la Lucha Contra las Enfermedades Neurológicas de la Infancia (FLENI), Ciudad Autónoma de Buenos Aires C1121A6B, Argentina; gvidela@fleni.org.ar

**Keywords:** baculovirus expression vectors, golden gate assembly, bio-inputs, gene therapy

## Abstract

Baculoviruses are insect-specific pathogens widely used in biotechnology. In particular, the Autographa californica nucleopolyhedrovirus (AcMNPV) has been exploited as a platform for bio-inputs production. This is why the improvement of the technologies used for the production of recombinant baculoviruses takes on particular relevance. To achieve this goal, we developed a highly versatile baculoviral transfer vector generation system called PluriBAC. The PluriBAC system consists of three insert entry levels using Golden Gate assembly technology. The wide availability of vectors and sticky ends allows enough versatility to combine more than four different promoters, genes of interest, and terminator sequences. Here, we report not only the rational design of the PluriBAC system but also its use for the generation of baculoviral reporter vectors applied to different fields of biotechnology. We demonstrated that recombinant AcMNPV baculoviruses generated with the PluriBAC system were capable of infecting *Spodoptera frugiperda* larvae. On the other hand, we found that the recombinant budded virions (BV) generated using our system were capable of transducing different types of tumor and normal cells both in vitro and in vivo. Our findings suggest that the PluriBAC system could constitute a versatile tool for the generation of insecticide and gene therapy vectors.

## 1. Introduction

The Baculoviridae family is composed of viruses that infect arthropods of the orders Lepidoptera, Hymenoptera, and Diptera [1] in larval stages. Baculoviruses have circular, double-stranded DNA genomes of 80 to 180 kbp, packed inside a rod-shaped nucleocapsid. The size of this nucleocapsid is 40–50 nm in diameter and 200–400 nm in length, and it is enveloped in a membrane derived from the host cell [2].

The nucleocapsid is incorporated into two different types of virions, depending on the stage of its life cycle. Outside the host, the virions are found embedded in a protein matrix forming the characteristic occlusion bodies (OBs) that protect the viruses from environmental conditions such as UV radiation. The viral cycle of baculoviruses starts when a susceptible insect feeds on foliar material containing OBs, and the protein matrix disintegrates in the midgut of the insect due to the change in pH, releasing the occlusion-derived viruses (ODVs) that infect the epithelial cells of the larval midgut via a membrane fusion mechanism [2]. The infected cells produce a second type of virion, a designated budded virus (BV), which spreads infection within the body of the insect larva [3].

Since the genes that code for the Polh (polyhedrin) and P10 proteins are not necessary for the transmission of BV in cell cultures and are expressed at very high levels during infection due to their very strong promoters, they have been used for the development of expression vectors. The coding region of these proteins, after being replaced with heterologous genes, resulted in the production of high levels of the protein of interest [4,5]. The various technological improvements developed to simplify production procedures have made the baculovirus/insect cell system one of the most widely used strategies [6]. Unlike prokaryotic systems, the baculovirus expression system provides many of the required post-translational modifications (folding, disulfide bonding, oligomerization).

The baculovirus expression vector system (BEVS) has been around since the early 1980s. Originally, these insect-specific pathogens were attractive because of their usefulness as biological pest control agents, posing as an alternative to chemical insecticides in the agroindustry. However, with the successful production of recombinant human IFN-β [7], by employing genetically modified Autographa californica multiple nucleopolyhedrovirus (AcMNPV) and lepidopteran cells, BEVS positioned itself as a highly versatile and powerful tool in biotechnology. To this day, thousands of proteins have been expressed in this system and some of them even became commercially available [2].

BVs can also enter into mammalian cells and transduce DNA [8]. The coding sequence for the gene of interest can be included in the viral genome under the control of a suitable promoter recognized with the target organism [9]. One of the subsequent inoculations with the recombinant BV results in the expression of the heterologous protein within different cell types [2]. BVs are safer than other transducing vectors as they do not replicate in vertebrate cells and integration of their DNA into the genome of host cells has not been observed. In addition, humans do not possess antibodies or specific T cells against BVs, unlike other transduction vectors that must overcome the obstacle of pre-existing immunity [10]. Baculoviruses have been modified using genetic engineering in order to facilitate the generation of recombinants. One of the strategies consists of the use of a bacmid, or viral genome, capable of replicating in a bacterial cell, but with a gene deletion that impairs its replication in insect cells, and a plasmid transfer vector that includes the gene of interest, and the viral gene that is missing in the bacmid. Then, recombinant AcMNPV genomes can be obtained using homologous recombination in insect cells [11,12] or transposition in bacteria [13].

Golden Gate assembly is based on the ability of type IIS restriction enzymes to cleave DNA outside their recognition site. This approach can be exploited to obtain specific 4-bp selected complementary overhanging sequences after digestion, which allow the simultaneous assembly of multiple fragments in one destination vector in a single restriction-ligation step [14]. In recent years, this cloning method has been used to develop different systems of multigene constructs to be applied in diverse organisms such as plants [15,16], fungi [17], or yeast [18,19]. Furthermore, Neuhold and colleagues developed a Golden Gate-based system, designated GoldenBac, to construct two sets of vectors to express up to 15 gene expression cassettes in the baculovirus [20].

In this study, we describe a versatile and rapid system based on the Golden Gate cloning method to express heterologous genes in baculoviruses. The PluriBAC system was challenged using diverse biotechnological platforms with successful results. Finally, we present a comparison between PluriBAC and other Golden Gate-based systems, focusing on the current methods applied in the baculovirus. The PluriBAC system stands as the pioneering Golden Gate-based method for generating recombinant baculoviruses with the required versatility to be adapted to various technological platforms and diverse fields within biotechnology.

## 2. Materials and Methods

### 2.1. PluriBAC System Design and Vector Building

The different recombinant vectors and products in the following text are identified as levels 0, 1, and 2. For level 0, specific primers were designed to obtain the different individual expression cassettes. In all cases, PCRs were prepared with Q5^®^ High-Fidelity DNA polymerase (New England Biolabs, Ipswich, MA, USA), and PCR products were purified using a gel extraction kit (PB-L), following manufacturer’s instructions. All modules were flanked by Esp3I restriction sites and specific sticky ends. To proceed in Golden Gate assembly, purified products were directly added to the mix or previously cloned into an entry vector, when appropriate.

For level 1, pGGL1 backbone was designed based on pUC57 mini vector (AmpR) including a cytotoxin-encoding *ccdB* gene as a positive selection marker. The *ccdB* gene was flanked by two Esp3I sites and the specific overhang sequences to accept modules from level 0. Furthermore, the fragment was also flanked by two BsaI sites and specific 4 bp overhang to act as a donor vector for cloning in level 2.

With regard to level 2, pGGL2Bac acceptor vector was designed based on pUC57 plasmid (Kan) containing suicide *ccdB* gene, two BsaI sites, and specific 4 bp overhang sequences. Furthermore, this vector contained the required sequences to allow the generation of recombinant baculovirus using the GOZA system [11,12]. Synthetic sequences were obtained from GenScript Inc. (Piscataway, NJ, USA).

### 2.2. Golden Gate Assembly Protocol

Golden Gate reactions were prepared by mixing 0.5 µL (30 U) of type IIS enzyme (BsaI or Esp3I), 1 μL (5U) of T4 ligase, 2 μL of 10× T4 ligase buffer with 100 ng of destination vector, and 200 ng of each insert in a final volume of 20 μL. Reactions mixtures were incubated 10 min at 37 °C and 15 min at 16 °C for 30 cycles and a final step of 5 min at 60 °C was carried out.

Then, 3 μL of the reaction mix was transformed into electrocompetent *E. coli* TOP10 cells and, after a 1 h incubation at 37 °C, cells were plated on LB agar supplemented with antibiotic. The screening to validate the inserts was assayed with colony PCR, digestion using traditional restriction enzymes, or both. In all cases, the inserts were validated using Sanger sequencing.

### 2.3. Insect Cells

Insect cell line *Trichoplusia ni* BTI-TN-5B1-4 (High Five^TM^ cells; Thermo Fisher Scientific, Waltham, MA, USA) was maintained in Grace’s medium (Thermo Fisher Scientific) supplemented with 10% of fetal bovine serum at 27 °C in T-flasks.

### 2.4. RecAcMNPV Generation

Recombinant pGGL2 vectors were co-transfected in insect High Five^TM^ cells with the occ+ bApGOZA bacmid DNA [11,12] using the lipid transfection reagent Cellfectin IITM (Thermo Fisher Scientific) following manufacturer’s instructions. Cells were maintained in Grace’s medium supplemented with 10% fetal bovine serum at 27 °C in T-flasks until signs of infection became apparent.

For Bac-to-Bac^®^ expression system, pGGL1 recombinant vectors containing Tn7R and Tn7L sequences were used to transform *E. coli* D H10Bac^TM^ electrocompetent cells. Transformed cells were plated in LB medium containing Kanamycin (50 µg/mL), Tetracycline (10 µg/mL), and any other antibiotic required for the selection. IPTG (40 µg/mL) and X-gal (200 µg/mL) were used for blue/white selection of the colonies in which transposition occurred. Recombinant bacmid was extracted following manufacturer’s indications from white colonies and used to transfect High Five^TM^ cells with the lipid Cellfectin II^TM^ reagent (Thermo Fisher Scientific) following manufacturer’s instructions. Cells were maintained in Grace’s medium supplemented with 10% of fetal bovine serum at 27 °C in T-flasks until signs of infection became apparent.

### 2.5. BV Titration

BVs were titrated on High Five^TM^ cell monolayers as plaque forming units (PFU); these titers were coincident with infectious foci as determined with fluorescence microscopy.

### 2.6. RecAcMNPV Per Os Infection

#### 2.6.1. RecAcMNPV Occlusion Bodies Extraction

Extraction of occlusion bodies (OBs) was performed from infected High Five^TM^ cells. Briefly, infected cells were resuspended and centrifuged 5000× *g* for 15 min. Supernatant was discarded and 3 mL of lysis buffer (10 mM Tris-Hcl pH 7,4, 10 mM EDTA, 0.25% SDS) was added to the pellet and incubated at 37 °C for 30 min. Suspension was centrifuged 350× *g* for 15 min to pool down cellular debris. Supernatant containing OBs was transferred to another tube and centrifuged at 17,000× *g* for 15 min. The pellet was resuspended in PBS and the presence of OBs was verified under light microscope. Quantification of OBs was performed using the Neubauer chamber as described in Eberle et al., 2012 [21].

#### 2.6.2. Spodoptera Frugiperda Larvae Per Os Infection

Third instar *Spodoptera frugiperda* larvae were per os infected with recAcMNPV coding the fluorescent protein dTomato. For infection, insects were allowed to feed on an artificial diet previously contaminated with a suspension of 3 × 10^8^ OBs/mL (3 × 10^6^ OBs in total per diet fraction according to doses reported previously by Pantha et al. [22]) or on a virus-free diet, for the control group. Larvae were kept individually in plastic cups, incubated at 27 °C, 16:8 h L:D, and monitored daily for signs of infection. Infected and uninfected larvae were observed with epifluorescence microscopy at 4 dpi.

### 2.7. Mammalian Cells

Primary cultures of mouse astrocytes obtained from the brain cortex were grown in Petri dishes previously coated with gelatin containing DMEM with high glucose, L-glutamine, and sodium pyruvate supplemented with 10% FBS and 1% penicillin-streptomycin. Cells were harvested using trypsin-EDTA (0.05%) and were counted with Trypan-Blue.

Human adenocarcinoma alveolar basal epithelial cells A549 were cultured in DMEM medium containing 10% FBS. To collect the cells, 0.05% trypsin-EDTA was used, and then they were counted with Trypan-Blue.

G09 cells are patient-derived glioblastoma (GBM) cells previously obtained from glioma biopsies [23]. The use of these cultures for biomedical research was approved by the Ethics Committee in Biomedical Research of the Foundation for the Fight Against Neurological Diseases in Childhood (FLENI, Buenos Aires, Argentina). These cells were cultured on Geltrex-coated Petri dishes with serum-free neurobasal medium supplemented with glucose; sodium pyruvate; PBS-BSA 7.5 mg/mL; 1× B27; 1× N2, 20 ng/mL bFGF and EGF; 2 mM L-glutamine; 2 mM non-essential amino acids; and 50 U/mL penicillin/streptomycin. The cells were harvested with accutase and counted with Trypan-Blue.

### 2.8. Baculovirus-Mediated Transduction In Vitro

Murine astrocytes and GBM patient-derived G09 cells were transduced as we previously reported [24]. Briefly, cells were seeded in a 24-well plate and, after 24 h, were incubated with the recAcMNPV that encodes Citrine fluorescent protein (750 PFU/cell) for 2 h in DMEM or Neurobasal medium, respectively. Finally, 0.25 mL of supplemented DMEM or Neurobasal medium was added.

A549 cells were seeded in a 12-well plate. When these cells were 60% confluent, they were incubated with 4 × 10^11^ PFU/mL of recAcMNPV (500 PFU/cell) encoding dTomato fluorescent protein under the regulation of pMDR1 (multidrug resistance protein 1 promoter). After a two-hour incubation period, 1 mL of MEM + 10% SFB was added per well.

### 2.9. Animals

Adult male C57Bl/6 mice (6–8 weeks old) were obtained from the animal facility of the Faculty of Veterinary Sciences, National University of La Plata, Argentina. Mice were maintained under controlled conditions of light (12 h light/dark cycles) and a temperature of 20–25 °C. They were fed standard feed and water ad libitum and their environment was cared for to minimize stress. All animal experimentation was performed under the guidance of the NIH and was approved by the Institutional Committee for the Care and Use of Laboratory Animals (CICUAL), School of Medicine, University of Buenos Aires; Res. (CD) No. 697/19.

### 2.10. Baculovirus-Mediated Transduction In Vivo

In order to characterize the transduction capacity of the vectors in vivo, mice were injected using stereotactic surgery in the brain with Citrine encoding recAcMNPV (5 × 10^8^ PFU/5 μL). After 7 days, mice received an overdose of ketamine/xylazine and were perfused with heparinized Tyrode’s solution and 4% paraformaldehyde (PFA). Brains were removed and fixed with 4% PFA for an additional 48 h, resuspended in cold 20% sucrose for 16 h, and finally frozen in an acetone–isopentane bath at −80 °C. Cryostat sections of 50 μm were obtained, and transduction efficiency was subsequently analyzed by observing the expression of the reporter gene Citrine.

To assess the ability of BV to transduce glioma cells in vivo, we intracranially inoculated mouse GBM neurospheres [25] into the right striatum of naive mice (n = 3). Three weeks later, we injected recAcMNPV intratumorally (5 × 10^8^ PFU in 5 μL), and seven days later mice were perfused with heparinized Tyrode’s solution and 4% PFA. Brains were processed, as described above, to assess transduction efficiency by observing the expression of the reporter gene Citrine.

### 2.11. Cisplatin pMDR1 Induction

A549 human lung cancer cells, in a 60% confluence state, were transduced with 4.4 PFU/mL of the recAcMNPV that encodes dTomato fluorescent protein under the regulation of Multidrug Resistance Protein 1 promoter (pMDR1). Two hours after transduction, we added cisplatin to the medium at different concentrations as follows: 6 µg/mL, 7.5 µg/mL, 9 µg/mL, and 10 µg/mL [26,27,28,29,30]. pMDR1 induction and dTomato expression were monitored daily with epifluorescent microscopy. To analyze the results, we counted the total cells and dTomato (+) cells with ImageJ software v1.52p. For statistical analysis, Student’s *t* test was performed. Data were graphed and analyzed using GraphPad Prism version 8.00 software (GraphPad Software Boston, MA, USA). Differences between groups were considered significant when *p* < 0.001. All the experiments were performed at least twice.

### 2.12. Epifluorescence Microscopy

Recombinant protein expression was verified with epifluorescence microscopy Nikon Eclipse Ti-S (Nikon Instruments Inc. Melville, NY, USA) and photographed with Nikon DS-Ri2 camera. Images were processed with ImageJ software.

## 3. Results

### 3.1. PluriBAC as a Versatile Multi Entry Level System

**Figure 1:**
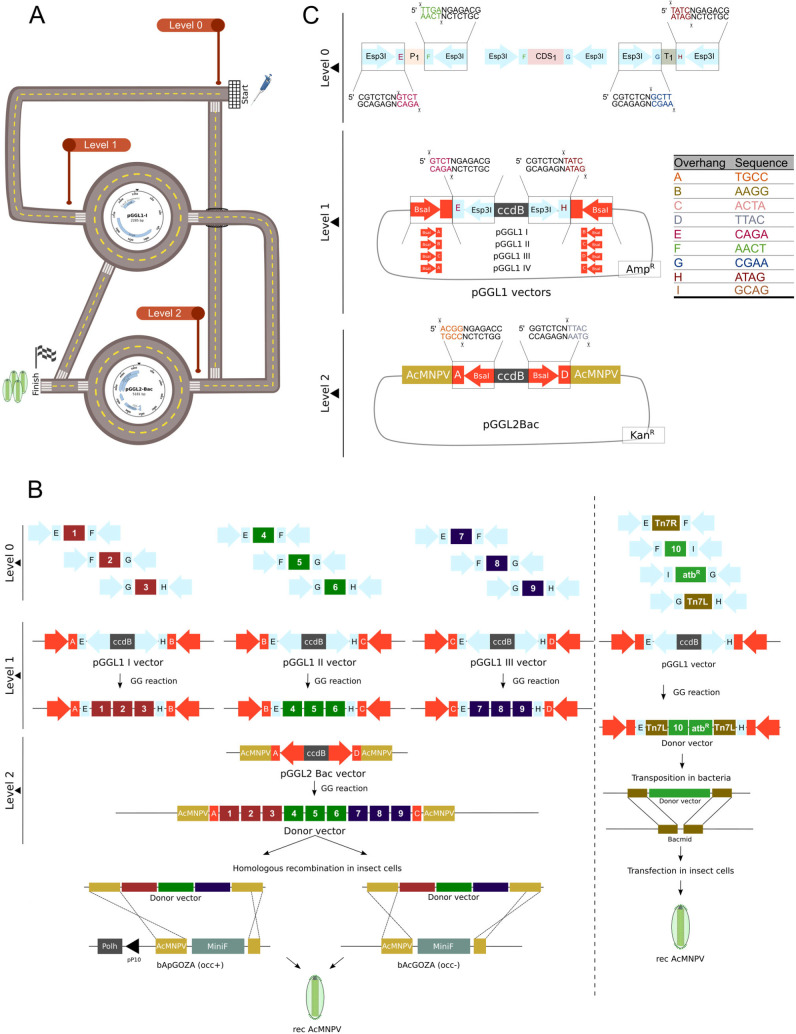
Schematic representation of PluriBAC system. (**A**) A hypothetical route that simulates the different pathways that can be taken to the generation of recAcMNPV with PluriBAC system. (**B**) Left: workflow describing the generation of recAcMNPV using homologous recombination (HR) between PluriBAC transfer vectors and the occ+ bApGOZA bacmid or the occ- bAcGOZA bacmid, from level 0 to level 2, going through level 1, where AcMNPV in yellow box represents the required sequence for the rescue of recombinant baculoviruses with HR. Right: generation of recAcMNPV using Bac-to-Bac strategy from a donor vector obtained with PluriBAC system. (**C**) A detailed scheme of the modules and vectors that are used in all three levels of the system. PCR-amplified inserts containing Esp3I restriction sites (light blue arrows) and specific overhangs are assembled in acceptor vectors from level 1 (pGGL1 I-IV) in one Golden Gate reaction. Resulting level 1 vectors contain the expression cassettes flanked by BsaI restriction sites (orange arrows) and specific overhangs in a strategy that permits the orderly assembly of all fragments in the final level 2 acceptor vector (pGGL2Bac) in one reaction involving BsaI RE. A table detailing the 4-bp overhangs used in this system is included.

The PluriBAC system is based on the Golden Gate assembly strategy, which allows for the simultaneous insertion of multiple fragments into one vector, utilizing type IIS restriction enzymes. PluriBAC comprises three levels and enables the generation of recombinant baculovirus from both level 1 and level 2 (Figure 1A). Level 0 consists of DNA inserts flanked by Esp3I sites and specific overhangs. Destination level 1 vectors contain the *ccdB* suicide gene flanked by Esp3I restriction sites and 4-bp overhangs compatible with level 0 inserts. To assemble the resulting expression cassettes into the final pGGL2Bac vector, level 1 vectors also contain BsaI restriction sites, in addition to an ampicillin resistance cassette. Level 2 acceptor vectors consist of a *ccdB* suicide gene flanked by BsaI restriction sites, with overhangs compatible with the resulting level 1 modules, and a kanamycin resistant cassette. After a Golden Gate reaction using an Esp3I enzyme, inserts from level 0 will contain unique overhangs compatible with level 1 vectors. Hence, they will assemble in an orderly manner to conform the expression cassettes in level 1 destination vectors. In the same way, the cleavage of the resulting level 1 vectors with BsaI allows their orderly assembly in the final pGGL2Bac vector. Furthermore, a fourth level 1 vector was designed to be used when only two inserts need to be incorporated to the final vector pGGL2Bac. Also, the level 2 destination vector has the AcMNPV sequence required for the homologous recombination to produce recombinant baculoviruses with the GOZA system. Although our system consists of three levels, from 0 to 2, the versatility of the platform allows the cloning from level 0 to level 2 (bypassing level 1) and also the direct generation of recombinant baculovirus from level 1, using the Bac-to-Bac strategy, as required (Figure 1B,C). Finally, the nine specifically designed different 4-bp overhangs were marked from “A” to “I”, and are detailed in Figure 1C.

### 3.2. PluriBAC-Derived recAcMNPV Could Carry on Larvae Per Os Infection

**Figure 2:**
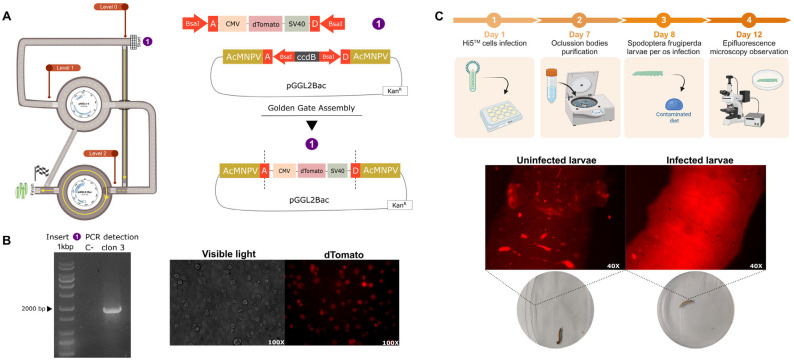
Per os infection of *Spodoptera frugiperda* larvae with PluriBAC-derived recAcMNPV. (**A**) A representation of the pathway followed in PluriBAC scheme to construct the virus, and the final pGGL2Bac donor vector obtained to generate the recAcMNPV. CMV represents cytomegalovirus immediate-early gene 1 (CMV-IE1) promoter and SV40 refers to simian virus 40 polyadenylation signal (SV40 polyA). (**B**) PCR screening of the insert: CMV + dTomato + SV40 (1650 bp) and fluorescent infected High Five^™^ cells. (**C**) Experimental protocol (created with BioRender.com, accessed on 14 September 2023) and epifluorescence microscopy of infected and uninfected *Spodoptera frugiperda* larvae with recAcMNPV.

To explore the ability of PluriBAC-derived recAcMNPV to infect *Spodoptera frugiperda* larvae per os, we generated a recombinant baculovirus encoding the reporter protein dTomato under the control of the CMV IE-1 promoter and SV40 polyA signal. We amplified the whole cassette using PCR and assembled it in the vector pGGL2Bac (Figure 2A). Insertion of the fragment was screened using PCR (Figure 2B) and confirmed with Sanger sequencing. The success in recAcMNPV generation was evidenced with epifluorescence microscopy on co-transfected High Five cells^TM^ (Figure 2B). High Five^TM^ cells were infected with the recAcMNPV and 7 dpi occlusion bodies (OBs) were purified from the cell culture using the method described previously. Third instar *Spodoptera frugiperda* larvae were infected per os with 3 × 10^8^ OBs/mL of recAcMNPV coding dTomato, by contaminating the diet with recOBs. Four days later, infected and uninfected larvae were observed with epifluorescence microscopy. Expression of dTomato was observed only in the larvae infected with the recombinant baculovirus (Figure 2C). The experimental pipeline is shown in Figure 2C.

### 3.3. PluriBAC-Derived recAcMNPV Efficiently Transduce CNS Cells In Vitro and In Vivo

**Figure 3:**
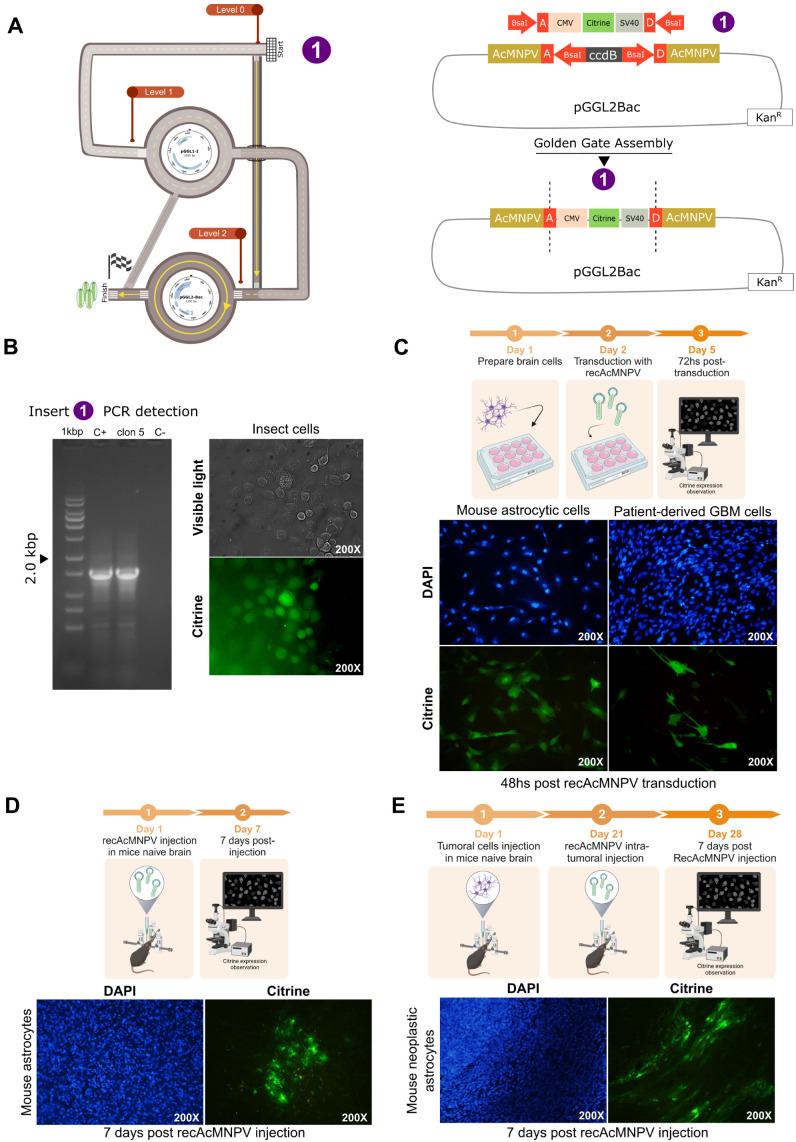
CNS cells transduction with PluriBAC-derived recAcMNPV. (**A**) A representation of the pathway followed in PluriBAC workflow to generate the virus, where CMV stands for cytomegalovirus immediate-early gene 1 (CMV-IE1) promoter and SV40, for simian virus 40 polyA signal (SV40 polyA). (**B**) PCR screening of the expression cassette (1485 bp) and fluorescent High Five^™^ cells infected with the recAcMNPV obtained. (**C**) Experimental protocol (created with BioRender.com, accessed on 14 September 2023) and epifluorescence microscopy of mice astrocytes and patient-derived GBM cells transduced with recombinant baculovirus. Citrine expression was observed 48 h after transduction. (**D**) Experimental protocol (created with BioRender.com, accessed on 14 September 2023) of the in vivo assay and citrine expression observation of mice normal astrocytes. C57Bl/6 mice were inoculated into the right striatum with BV (5 × 10^8^ PFU). After 7 days, the expression of the reporter protein citrine (green) was evaluated with fluorescent microscopy. (**E**) Experimental protocol (created with BioRender.com, accessed on 14 September 2023) of the in vivo assay and citrine expression observation of mouse neoplastic astrocytes. C57Bl/6 mice were inoculated with mouse glioma neurospheres (NS) into the brain; 3 weeks later, tumors were injected with BV (5 × 10^8^ PFU) and mice were euthanized after 7 days. The expression of the reporter protein citrine (green) in the tumor was evaluated with fluorescent microscopy. In all cases, DAPI was used for nuclear staining.

In order to assess the capacity of PluriBAC-derived recAcMNPV to transduce mammalian central nervous system (CNS) cells, we generated a recombinant baculovirus encoding the green fluorescent protein Citrine under the control of the CMV IE-1 promoter and SV40 polyA signal fragment, which was confirmed using PCR (Figure 3B) and Sanger sequencing. Successful recAcMNPV generation was evidenced with epifluorescence microscopy on co-transfected High Five cells^TM^ (Figure 3B).

With the aim to evaluate the transduction performance of recAcMNPV, we transduced both mice astrocytic cells and patient-derived GBM cells in vitro. PluriBAC-derived recAcMNPV efficiently transduced both cell types at MOI of 750, leading to robust Citrine expression (Figure 3C). Please note that for practical purposes, MOI (multiplicity of infection) is used here to indicate infective virus particles (PFU) per cell used, regardless of the fact that no baculovirus infectious cycle proceeds in mammalian cells.

Finally, we evaluated the ability of recAcMNPV to transduce CNS cells in vivo. To address this issue, we developed two experimental approaches using animal models.

Seven days after injection into the striatum of mice using stereotactic surgery with 5 × 10^8^ PFU of Citrine encoding recAcMNPV, reporter gene expression was observed in normal astrocytes (Figure 3D).

On the other hand, we assessed recAcMNPV transduction capacity on mouse neoplastic astrocytes. We injected mice with mouse GBM neurospheres using stereotactic surgery. After 21 days, we treated mice with recAcMNPV, as described before [24]. PluriBAC-derived recAcMNPV encoding Citrine could effectively transduce mouse neoplastic astrocytes as assessed 7 days after injection (Figure 3E).

### 3.4. Chemotherapy-Inducible Promoters Can Also Be Chosen to Generate recAcMNPV for Gene Therapy

The ABC transporters (ATP-binding cassette), in particular, the multidrug resistance protein 1 (MDR1 or ABCB1) can resist cancer conventional treatment, such as chemotherapy. Through a bibliographic search, we found reports of elevated levels of MDR1 mRNA and protein in numerous clinical samples [31,32,33,34].

**Figure 4:**
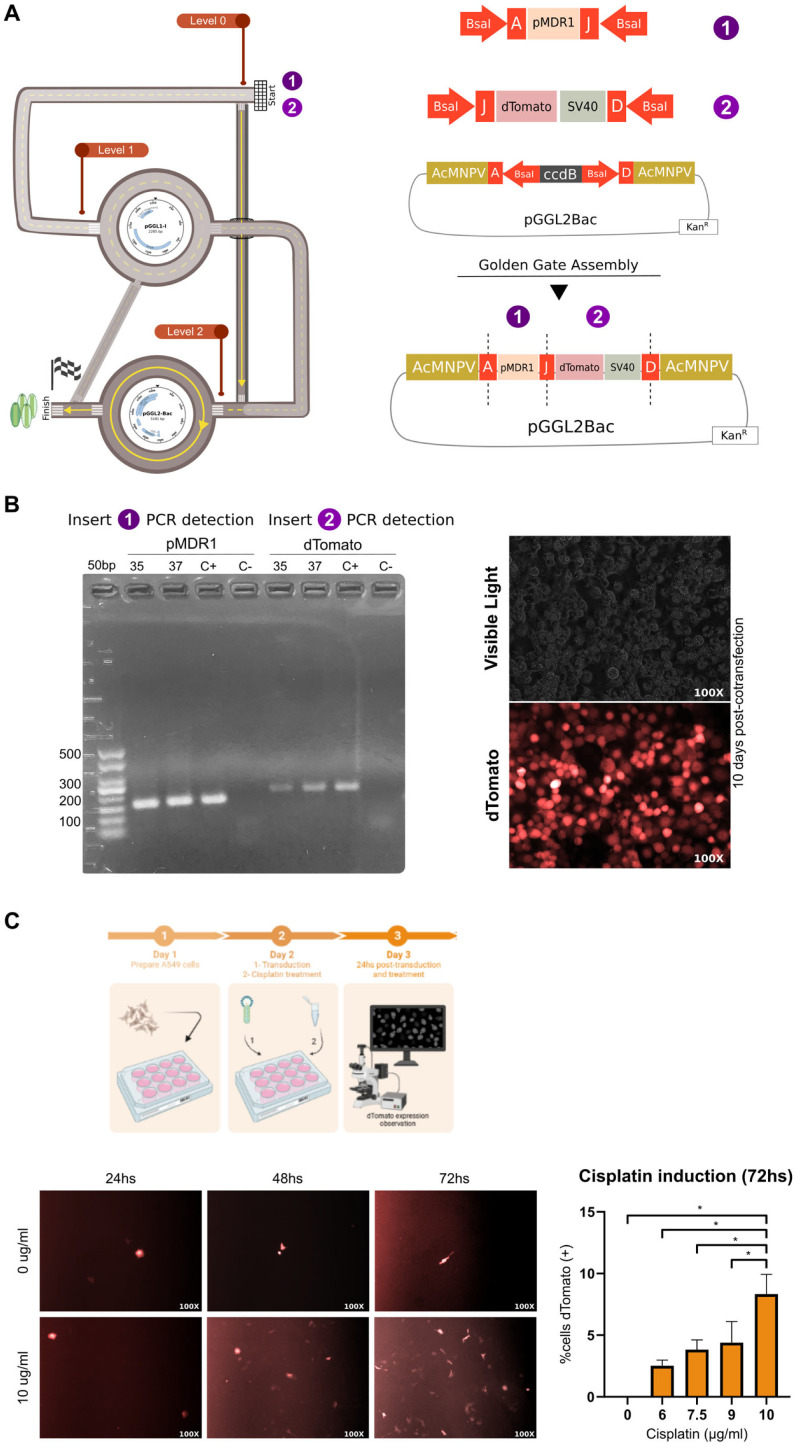
Characterization of the induction of pMDR1 with cisplatin using a PluriBAC-derived AcMNPV encoding dTomato fluorescent protein. (**A**) A representation of the pathway followed in PluriBAC workflow to generate the baculovirus. SV40: simian virus 40 polyA signal. (**B**) PCR screening of the inserts: pMDR1 (1393 bp), dTomato + SV40 (864 bp), and obtained fluorescent High Five™ cells infected with the recAcMNPV. (**C**) Experimental protocol (created with BioRender.com, accessed on 14 September 2023) and epifluorescence microscopy of transduced A549 cells treated with cisplatin at different times (for practical purposes, only two cisplatin concentrations are represented). Percentages of positive cells were calculated in the different cisplatin concentration assayed at 72 h post-transduction. A Student’s *t* test was performed between groups, * *p* < 0.001.

To characterize the induction of the MDR 1 promoter (pMDR1) with cisplatin, we designed a recAcMNPV that encodes dTomato fluorescent protein under the regulation of pMDR1 generated by the PluriBAC system (Figure 4A). To achieve this goal, we amplified two inserts using PCR with primers that added BsaI restriction sites and specific overhangs in order to allow their assembly into the pGGL2Bac vector (Figure 4A). The correct assembly of the vector was confirmed using PCR (Figure 4B) and Sanger sequencing.

We used double homologous recombination in High Five cells^TM^ for recAcMNPV generation, evidenced with epifluorescence microscopy of co-transfected insect cells (Figure 4B).

Finally, we transduced A459 human lung cancer cells with recAcMNPV at an MOI of 500. Two hours after transduction, we incubated the cells with different concentrations of cisplatin, i.e., 6 µg/mL, 7.5 µg/mL, 9 µg/mL, and 10 µg/mL, in order to detect how many hours post-transduction and which cisplatin concentration would better induce pMDR1 (Figure 4C).

We observed dTomato expression 48 h after transduction and cisplatin treatment. However, the best condition for pMDR1 induction was at 72 h post-transduction, in cells treated with 10 µg/mL of cisplatin. As a result, we detected a significantly greater number of cells expressing the fluorescent protein dTomato in this group compared with the control group, which was only transduced with recAcMNPV, but was not treated with cisplatin (Figure 4C).

### 3.5. Multiple Insert Assembly in Bac-to-Bac^TM^ Expression System

Bac-to-Bac^TM^ (Thermofisher) is a baculovirus expression system that allows users to construct recAcMNPV vectors by site-specific transposition between Tn7L and Tn7R sequences and the bacmid bMON14272, using a transposase present in the DH10Bac^TM^ strain.

**Figure 5:**
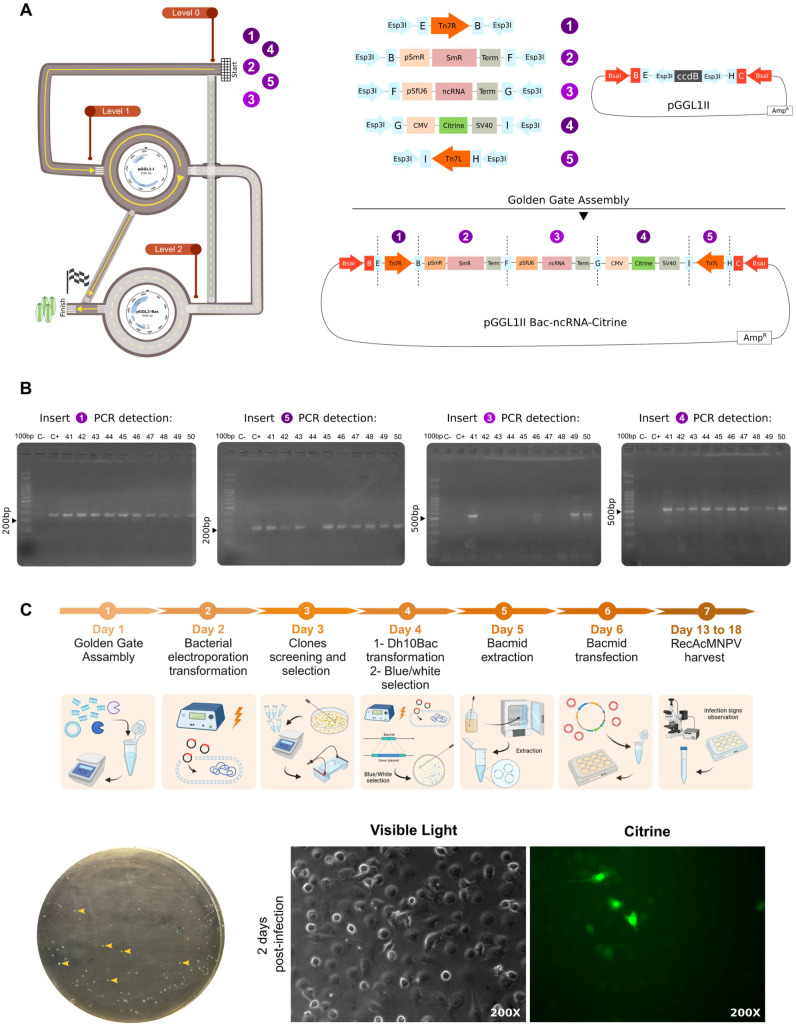
Multiple insert PluriBAC-derived recBV constructed using *Bac-to-Bac*^®^ expression system. (**A**) A representation of the pathway followed in PluriBAC scheme to construct the virus, and the final pGGL1-Bac donor vector obtained to generate the recAcMNPV with *Bac-toBac^TM^* expression system. Tn7L and Tn7R: transposition sites, pSpect: Spectinomycin resistance promoter, Spect R: Spectinomycin resistance, pSfU6: small nucleolar U6 *Spodoptera frugiperda* promoter, ncRNA: synthetic non-coding RNA, CMV: cytomegalovirus immediate-early gene 1 (CMV-IE1) promoter, SV40: simian virus 40 polyA signal. (**B**) PCR screening of the multiple inserts. Insert 1: Tn7R (260bp), insert 3: pSfU6 + ncRNA + term (520 bp), insert 4: CMV (507 bp), insert 5: Tn7L (211 bp). Spectinomycin resistance and its promoter were screened by using Spectinomycin for clonal selection. (**C**) Experimental protocol (created with BioRender.com, accessed on 14 September 2023) of recAcMNPV generation using Bac-to-Bac^TM^ system. Blue–white transposition screening in *E. coli* DH 10Bac^TM^ cells and epifluorescence microscopy of infected High Five^TM^ cells with recBV. Yellow arrows show some of the white colonies obtained.

We constructed a pGGL1 vector flanked by Tn7R and Tn7L sequences to obtain the recAcMNPV with the Bac-to-Bac^TM^ system (Figure 5A). We also included a spectinomycin resistance gene, an ncRNA under the control of a SfU6 promoter, and a reporter gene *citrine* under the control of the CMV-IE-1 promoter and the SV40 polyA signal. Inserts were amplified using PCR with specific primers containing Esp3I restriction sites and cohesives. After Golden Gate assembly, *E. coli* DH 5α cells were transformed using electroporation and plated in LB medium with ampicillin, spectinomycin, and tetracycline. Inserts were screened using PCR, with the exception of spectinomycin resistance that was selected with the antibiotic. Most of the screened clones contained all the fragments, as can be appreciated in Figure 5B. Identity of the fragments was checked with Sanger sequencing.

After pGGL1-Bac-ncRNA-Citrine was constructed, *E. coli* DH10Bac^TM^ electrocompetent cells were transformed using electroporation and grown in LB medium with kanamycin, spectinomycin, and tetracycline, and X-gal and IPTG for blue/white screening (Figure 5C).

White colonies were selected and the recombinant bacmid was extracted following the manufacturer’s instructions. Integrity was checked with electrophoresis in 0.8% agarose gel stained with Ethidium Bromide. Then, Sf9 cells were transfected with the recombinant bacmid using a Cellfectin II^TM^ lipid reagent (Thermo Fisher Scientific) and following the manufacturer’s instructions. recAcMNPV generation was followed with epifluorescence microscopy to identify reporter gene citrine expression (Figure 5C).

## 4. Discussion

Since the 1980s, the baculovirus expression vector system (BEVS) has become a pivotal biotechnological tool for the generation of recombinant proteins. The production of large amounts of protein is possible because of the insertion of the heterologous gene (or series of genes) under the control of a very late viral gene promoter, usually the polyhedrin gene (*polh*) promoter. It triggers a high expression of the gene of interest in the insect cells infected with the recombinant baculovirus. One extra benefit of BEVS involves the capacity of insect cells to facilitate glycosylation and other post-translational modifications in the target protein [35]. Currently, the following two main strategies are used for the generation of recombinant baculoviruses: one involves a site-specific transposition in *E. coli* while the other one is based on homologous recombination in insect cells, as mentioned before [2].

Recently, there has been an increasing need for the generation of multi-gene expression constructs. However, the assembly of multiple genes can be a laborious procedure that involves many steps. Therefore, different strategies have been developed to face the demand and some of them make use of the baculovirus. In 2004, Berger and colleagues created the MultiBac system to generate a recombinant baculovirus that expressed protein complexes comprising many subunits, based on Cre-loxP technology [36]. Many years later, the biGBac system, utilizing Gibson’s cloning strategy and the insertion of specific linker sequences, was used to assemble up to 25 genes into one baculoviral expression vector. Then, recombinant baculoviruses were obtained using the Tn7 transposition method [37].

In search of improved efficiency and more simplicity in the assembly of multiple genes, the significance of Golden Gate cloning has been growing in recent years and different systems based on this technology were developed. For example, a modular system, consisting of three levels, was designed for the assembly of multiple transcription units in acceptor backbones optimized for plant transformation [15]. Recently, Occhialini and colleagues adapted this strategy for chloroplast engineering in plants [38]. In addition, a Golden Gate-based system was used for the construction of polycistronic expression vectors assayed in tobacco leaves [16]. In 2021, a strategy based on the Golden Gate cloning method was designed with the aim to construct a library for the study of violacein, an anticancer therapeutic compound, in *Yarrowia lipolytica*, an oleaginous yeast [39]. Using a similar approach, another system was created to delete and insert genes in various fungi [17].

The GoldenBac system is a novel method based on GoldenGate cloning that consists of two sequential levels for the assembly of up to 15 gene expression cassettes with high efficiency. In short, GoldenBac consists of sets of entry vectors for the insertion of the genes of interest that contains a polyhedrin promoter and a terminator flanked by BsaI restriction sites. After cleavage, the release of expression cassettes with specific 4-bp overhangs permits the ordered assembly of the resulting modules in destination vectors of step 2, which is designed to allow the generation of recombinant baculoviruses using homologous recombination or Tn7 transposition [20].

Here, we present a versatile system based on Golden Gate cloning, designated PluriBAC, which is efficient for the rapid generation of recombinant baculoviruses for a wide range of applications. As schematized in Figure 1, a part of the versatility of the PluriBAC system lies in the fact that its design can lead to transfer vectors compatible with both systems that use bacmids based on homologous recombination (occ- bAcGoza and occ+ bApGoza), as well as systems based on transposition in bacteria (Bac-to-BacTM). The flexibility of being able to choose between different bacmids is relevant in relation to biosafety. If it is necessary to use some recAcMNPV outside the controlled environment of the laboratory, where occ- viruses could be generated both with HR and with transposition. The case of the presence of resistance to microbial agents in the recAcMNPV genome is similar to this. Recombinant genomes derived from bAcGoza and bApGoza bacmids do not retain antibiotic resistance after recombination, unlike the Bac-to-Bac system. This could be useful when the budding virions of recAcMNPV are used as therapeutic vectors. With this in mind, the most appropriate bacmid could be chosen in relation to the occlusion phenotype and the presence or absence of antibiotic resistance depending on the needs and biosafety standards required in each field or application.

Next, we generated a set of different recombinant baculoviruses as examples suitable for testing in diverse biotechnological platforms, including insect or mammalian cells, as well as insect larvae or mice.

First, we developed a recAcMNPV encoding the reporter gene dTomato with the aim of exploring its ability to infect lepidopteran larvae. We carried out a pilot infection test on larvae of the fall armyworm *Spodoptera frugiperda,* which has been shown to be resistant to infection with AcMNPV and requires high doses of this virus to be infected [22]. *Spodoptera frugiperda* (Lepidoptera: Noctuidae), is an invasive agricultural pest that severely affects a variety of crops and is naturally distributed across the Americas. Recent studies have also described the presence of the fall armyworm in Africa and Asia [40,41]. While traditional control methods involve the use of chemical products or crops expressing Bt toxins [42], more and more cases of resistance breakouts to Bt toxins and the environmental impact of chemical pesticides are pushing the development of novel control strategies [43,44]. Many efforts have been made to learn more about the interaction between this lepidopteran pest and its natural pathogens with the ultimate goal of developing new biocontrol strategies [44,45,46,47]. Consequently, a quite versatile AcMNPV recombinant generation system, such as PluriBAC, is of particular interest for progressing the design of these strategies to test alternative functional proofs of concept in larvae.

Taking advantage of the transducing capacity of BVs [2,9], we set out to test recAcMNPV derived from the PluriBAC system in mammalian cells both in vitro and in vivo, as a second experimental technological platform. To achieve this goal, we designed and constructed two recombinant AcMNPVs that express the Citrine and dTomato reporter proteins, respectively. Based on our previous experience in baculovirus-mediated transduction of rat pituitary tumor cells [48], rat and human ovarian cancer cells [49], and rat, mouse, and human CNS cells [24,50], we explored the transducing ability of both recAcMNPVs. PluriBAC-derived recombinant baculoviruses efficiently transduce murine astrocytes, human adenocarcinoma alveolar basal epithelial cells, and patient-derived GBM cells in vitro. Furthermore, recAcMNPVs transduced normal and neoplastic astrocytes in vivo in the mouse brain. The development of a system such as PluriBAC, which facilitates the expression of multiple transgenes in different organisms, enables a more efficient workflow for the design and production of immunogens and targeted therapy vectors for both human and veterinary healthcare.

One of the distinctive points of our system compared to similar systems [17,20,36] is that none of the PluriBAC level 1 and level 2 vectors contain promoter sequences. Although this absence might appear to be a problem, since it is necessary to separately amplify the promoter in each gene assembly, it actually turns out to be one of the strengths of the PluriBAC system. The fact that the user can choose the promoter for each particular assembly allows a design of customized viral vectors according to the required needs and the problems to be solved. Indeed, as we reported in Section 3.4, we constructed a recAcMPV capable of expressing dTomato in response to increasing concentrations of the chemotherapeutic drug cisplatin. These results showed the utility of the PluriBAC system for the generation of inducible expression vectors, and in particular, vectors capable of being used for combined therapeutic schemes. This is particularly relevant in pathologies such as cancer, where the complexity of its nature and the emergence of resistance events to conventional therapies require the development of new therapies to complement them [51].

Another advantage of the PluriBAC system lies in its very design. As we describe throughout this work, PluriBAC is a three-level system that allows gene assembly using both type IIS restriction enzymes BsaI and Esp3I. Furthermore, most of the available overhang sequences are not found in PluriBAC vectors and can be chosen by including them in the primer design for level 0 PCR. All these features allow the user to enter the PluriBAC system in a multi-pathway and alternate the use of the type IIS restriction enzyme according to the sequence requirements of the genes to be cloned. Additionally, as we show in Section 3.5, compatible transfer vectors can be constructed for both the Bac-to-Bac^TM^ system and systems based on homologous recombination. Furthermore, in the construction presented in Section 3.5, multiple gene products with biological functionality in different organisms were included (resistance to Spectinomycin in bacteria and the reporter gene Citrine in insect and mammalian cells). This suggests that the PluriBAC system could have the potential to be used in the generation of recAcMNPVs that incorporate multiple gene products that, unlike the recAcMNPV that we generated here, can be expressed simultaneously in the same organism.

Table 1 summarizes the most outstanding features of PluriBAC. It also presents a comparison with the recently reported GoldenBac system. GoldenBac and PluriBAC are the first Golden Gate assembly-based systems designed to produce recombinant baculoviruses. While GoldenBac was originally developed for the expression of multimeric proteins in insect cells, PluriBAC was designed with the aim of having a versatile gene expression system directed to multiple fields of technological application. However, this does not imply that recombinant baculoviruses derived from each system cannot be adapted for application in other fields in the future. In this way, both systems are complementary and contribute to the vast toolbox of genetic engineering of baculoviruses. 

## 5. Conclusions

In this work, we have presented a versatile, multilevel, Golden Gate-based, recombinant baculovirus generation system designated PluriBAC. PluriBAC showed excellent performance in cloning multiple heterologous sequences into the AcMNPV genome. Depending on the nature of these heterologous sequences, the PluriBAC-derived vectors could lead to the generation of recAcMNPVs using homologous recombination in insect cells or transposition developed in bacteria. Recombinant baculoviruses generated with the PluriBAC system were successfully used to infect larvae per os, transduce mammalian cells in vitro, and naive and ill mice brains in vivo. This intrinsic capacity to freely combine multiple different sequences with biological activity in many diverse organisms makes PluriBAC a very useful toolbox for baculoviral biotechnology.

## Figures and Tables

**Table 1:** Comparative analysis of the main features of the PluriBAC and GoldenBac systems. HR: homologous recombination.

Golden Gate-Based BEV System Features	PluriBAC	GoldenBac [20]
Number of levels	Three	Two
Path between levels	Multiway	One way
	Variable	Linear
Promoters present in vectors	None	Polyhedrin promoter
Overhangs designed in	Primers	Vectors
Type IIS RE available to use	Two	One
Recombinant baculovirus system compatibility	HR and Bac-to-Bac^TM^	HR and Bac-to-Bac^TM^
Expression platform flexibility	Insect cells Larvae Mammalian cells Mice	Insect cells
Reported biotechnological applications to date	Per os larvae infection Normal and tumor cell transduction in vitro Normal and tumor cell transduction in vivo Chemotherapy-inducible expression	Multimeric proteins expression

## Data Availability

Data available upon request (mlpidre@biol.unlp.edu.ar).
